# MicroRNA miR-92a-3p regulates breast cancer cell proliferation and metastasis via regulating B-cell translocation gene 2 (BTG2)

**DOI:** 10.1080/21655979.2021.1924543

**Published:** 2021-06-03

**Authors:** Huang Jinghua, Zhou Qinghua, Chen Chenchen, Chen Lili, Xu Xiao, Wang Yunfei, An Zhengzhe, Lin Changxiu, Han Hui

**Affiliations:** a Department of Radiation Oncology, Affiliated Hospital of Yanbian University, Yanbian, Jilin, China; b Department of Pain Control, Zoucheng People ‘S Hospital, Shandong, China; c Department of Gynecology, Affiliated Hospital of Jining Medical University. Jining, Shandong, China; d Department of Anesthesiology, Huaiyin People’s Hospital, Huaiyin District, Jinan, Shandong, China; e Central Laboratory, Affiliated Hospital of Yanbian University, Yanbian, Jilin, China

**Keywords:** Breast cancer, miR-92a-3p, BTG2

## Abstract

MicroRNAs (miRNAs) dysregulation contributes to tumorigenesis, and it is reported that abnormal miR-92a-3p expression participates in multiple cancers’ occurrence and progression. This study focuses on miR-92a-3p’s functions and regulatory mechanism in breast cancer (BC). The current study proved miR-92a-3p expression was enhanced in BC tissues and cells, and its high expression was related to increased TNM stage and larger tumor size of BC patients. Functionally, transfection of miR-92a-3p mimics facilitated BC cell proliferation and metastasis, yet transfection of miR-92a-3p inhibitors functioned oppositely. In addition, BTG2 was verified as a direct miR-92a-3p target in BC cells. This research indicated that miR-92a-3p facilitates BC cell proliferation and metastasis through repressing BTG2 expression.

## GRAPHICAL ABSTRACT

**Figure:**
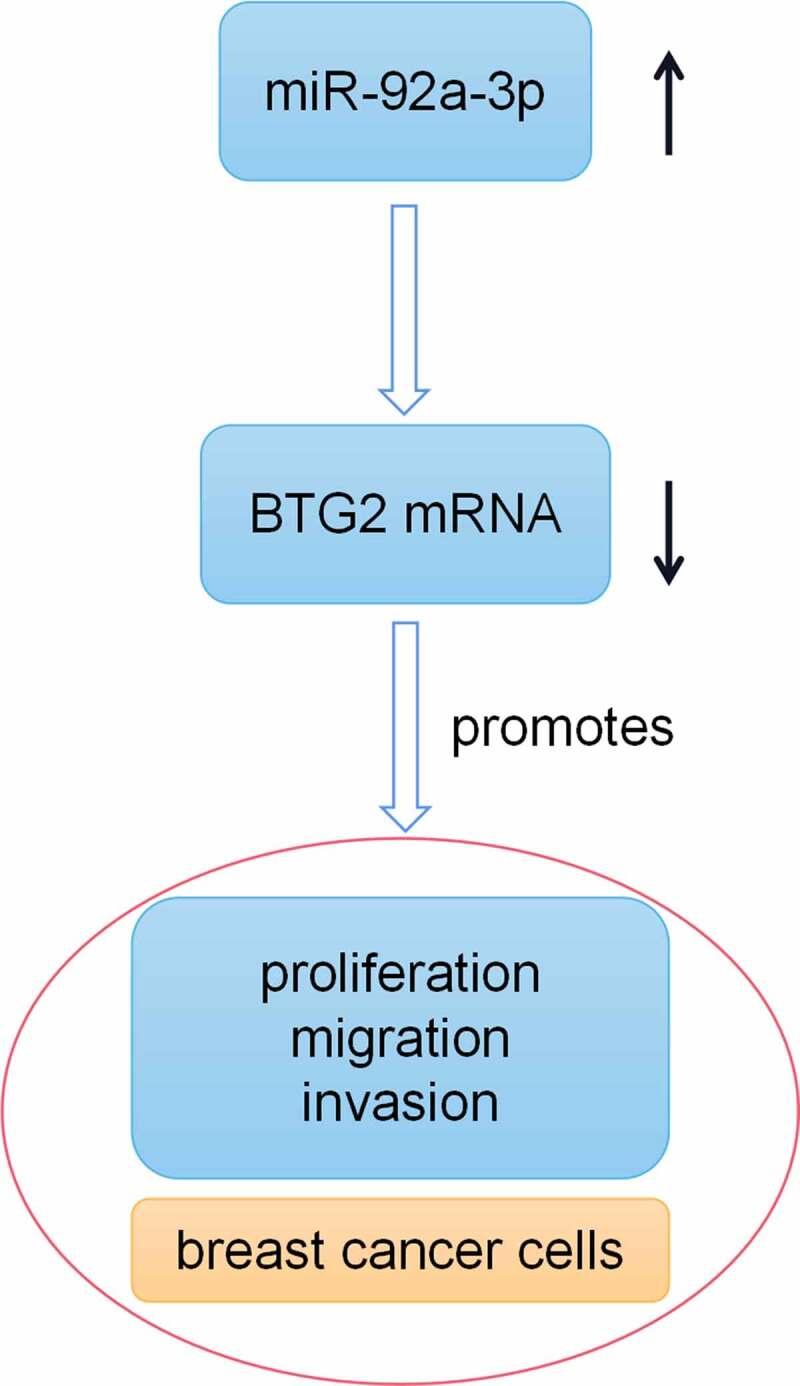

## Introduction

Recognized as one of most common malignancies in women, breast cancer (BC) makes up 11.6% of cancer cases and 6.6% of cancer-related deaths globally [1]. Though its clinical treatment strategies as well as its diagnosis have been significantly improved, high recurrence rate and drug resistance still limit the survival time of BC patients [2,3]. Elucidating BC’s molecular mechanism and discovering novel treatment targets are crucial for further increasing the survival time of BC patients.

Known as a kind of endogenous short non-coding RNAs, microRNAs (miRs or miRNAs) are approximately 20 nucleotides in length and can bind with mRNA at the 3ʹ-untranslated region (3ʹUTR), thereby partaking in modulating gene expression at the post-transcriptional level [4,5]. Multiple studies have supported that miRNAs, differently expressed in many cancers, are pivotal in a series of biological processes, e.g., cell growth, migration, invasion and apoptosis [6–8]. Also, previous research reports that miR-92a-3p facilitates the progression of various tumors. For instance, miR-92a-3p facilitates esophageal squamous carcinoma cell multiplication, migration and invasion by the regulation of phosphatase and tensin homolog (PTEN) [9]; miR-92a-3p functions as an oncomiR in glioma, and it promotes cancer cell proliferation via regulating Notch-1/Akt and CDH1/β-catenin signaling pathways [10]. Nonetheless, miR-92a-3p’s role and mechanism in BC development remain unclear.

Bioinformatics analysis suggest that miR-92a-3p and B-cell translocation gene 2 (BTG2) 3ʹUTR have potential complementary binding sites. BTG2 is identified as a member in the BTG/Tob anti-proliferation gene family [11]. BTG2, a tumor suppressor in various malignancies, is absent or down-regulated in multiple tumors, for instance, gastric carcinoma, hepatocellular carcinoma and non-small cell lung carcinoma [12–14]. Furthermore, BTG2 overexpression has been validated to repress BC cell proliferation and metastasis [15,16]. However, the mechanisms of miR-92a-3p/BTG2 axis in BC progression remain to be explored.

The present work validated that miR-92a-3p played a cancer-promoting role in BC, and further confirmed that BTG2 was a direct miR-92a-3p target. This study provides novel clues to the mechanism of BC tumorigenesis and progression.

## Materials and methods

### Tissue samples

The tissues used herein were collected from 60 BC patients in the Affiliated Hospital of Jining Medical University. (Table 1) shows the clinical information of the patients. The patients all signed an informed consent, and the experiments were endorsed by the Hospital’s Ethics Review Board. Following the *Declaration of Helsinki*, the study was conducted.

**Table 1.** Correlation of miR-92a-3p expression with multiple clinicopathological features in BC patients

Characteristics	Number (n = 60)	miR-92a-3p expression		χ^2^	P value
		Low	High		
Age (years)					
≤ 50	35	18	17	0.0686	0.7934
> 50	25	12	13		
TNM stage					
I–II	23	16	7	5.7109	0.0169
III–IV	37	14	23		
Histological differentiation					
High and moderate	27	13	14	0.0053	0.9419
Poor	33	17	19		
Tumor size					
≥ 5 cm	39	15	24	5.9341	0.0149
< 5 cm	21	15	6		
ER status					
Positive	33	15	18	0.6061	0.4363
Negative	27	15	12		
PR status					
Positive	35	19	16	0.6171	0.4321
Negative	25	11	14		
HER-2 status					
Positive	28	17	11	2.4107	0.1205
Negative	32	13	19		
Molecular subtype					
Luminal like	35	19	16	0.7238	0.6963
HER-2 positive	15	7	8		
Triple negative	10	4	6		

### Cell culture and transfection

From the American Type Culture Collection (Rockville, MD, USA), BC cell lines (MDA-MB-231, BT549, MCF-7 and BT474) as well as the normal mammary epithelial cell line MCF-10A were bought. Dulbecco’s Modified Eagle’s Medium (Invitrogen, Carlsbad, CA, USA) containing 10% fetal bovine serum (FBS; Gbico, Detroit, MI, USA) was utilized to culture the BC cells in 5% CO_2_ at 37°C in an incubator with saturated humidity. MCF-10A cells were cultured in RPMI-1640 medium (Invitrogen, Shanghai, China) containing 100 mg/ml streptomycin, 100 U/ml penicillin and 10% FBS (Invitrogen, Carlsbad, CA, USA).

miR-92a-3p mimics (5ʹ-UAUUGCACUUGUCCCGGCCUGU-3ʹ), mimics negative control (miR NC: 5ʹ-UUCUCCGAACGUGUCACGU-3ʹ), miR-92a-3p inhibitors (5ʹ-ACAGGCCGGGACAAGUGCAAUA-3ʹ) and inhibitors negative control (miR in: 5ʹ-UUGUCCGAACGUGUCACGU-3ʹ) were purchased from RiboBio (Guangzhou, China). BTG2 siRNA (si-BTG2: 5ʹ-GCUCCAUCUGCGUCUUGUA-3ʹ), siRNA negative control (si-NC: 5ʹ-UACGACCGGUCUAUCGUAG-3ʹ), BTG2 overexpression plasmids and empty vector (NC) were designed and synthesized by Generay Biotech (Shanghai, China).

MCF-7 and BT549 cells during logarithmic growth were trypsinized with 0.25% trypsin and inoculated in 6-well plates, and the cell density was adjusted to 1 × 10^5^ cells/well. When reaching a density of 50%-60%, the cells were washed twice with serum-free Opti-MEM (Invitrogen, Carlsbad, CA, USA). Besides, miR-92a-3p inhibitors (50 nM), miR-92a-3p mimics (50 nM), BTG2 overexpression plasmid (50 nM), si-BTG2 (50 nM), and respective negative controls (with a final concentration of 50 nM) were transfected into BT549 and MCF-7 cells using Lipofectamine 2000 (Invitrogen, Carlsbad, CA, USA) following the manufacturer’s instructions. Next, the aforementioned cells were cultured at 37°C for 24 h and then collected. Ultimately, the expressions of miRNAs or proteins were detected by quantitative real-time polymerase chain reaction (qRT-PCR) or Western blot.

### qRT-PCR analysis

TRIzol® reagent (Invitrogen, Carlsbad, CA, USA) was utilized for the extraction of total RNA from BC tissues and cells, and before qRT-PCR, ReverTra Ace qPCR RT Kit (Toyobo, Tokyo, Japan) was applied to reverse-transcribe 200 ng of extracted RNA into cDNA. qRT-PCR was conducted on a LightCycler 480 Real-time PCR system (Roche, Shanghai, China) with THUNDERBIRD SYBR® qPCR Mix (Toyobo, Tokyo, Japan). U6 and PPIA acted as endogenous control genes for normalizing target genes’ expression. The relative expressions of miR-92a-3p and BTG2 mRNA were calculated using the 2^−ΔΔCT^ method. The primer sequences are shown in (Table 2).

**Table 2.** Sequences used for qRT-PCR

miR-92a-3p	F: GCCGAGTATTGCACTTGTCC R: CTCAACTGGTGTCGTGGA
U6	F: CTCGCTTCGCRCAGCACA
	R: AACGCTTCACGAATTTGCGT
BTG2	F: CATCATCAGCAGGGTGGC
	R: CCCAATGCGGTAGGACAC
PPIA	F: GGTTCCCAGTTTTTCATTTG
	R: ATGGTGATCTTCTTGCTGGT

Abbreviations: F represents forward; R represents reverse; RT represents reverse transcription.

### Cell proliferation assay

Cell counting kit-8 (CCK-8; Dojindo Molecular Technologies, Rockville, Japan) was employed to evaluate cell proliferation. After 24 h of transfection, MCF-7 and BT549 cells were plated in 96-well plates (2000 cells/well, 100 μl) and cultured for 24 h at 37°C. Then, each well was added with 10 μL of CCK-8 solution at different time points (12, 24, 48, 72 and 96 h), and incubated at 37°C for 4 h. Ultimately, a microplate reader was utilized to determine the absorbance of cells in each well at 450 nm.

### Transwell assay

The procedures for migration assay and invasion assay were similar, except that Matrigel (30 μg/well; BD, San Jose, CA, USA) coating was adopted for invasion assay, but not in migration assay. BT549 and MCF-7 cells were re-suspended with serum-free medium and pretreated with 5 μg/ml mitomycin C (Sigma, St Louis, MO, USA) for 2 h to prevent cell proliferation. Cells were then inoculated in the top chamber of Transwell chambers (8 μm pore diameter, Corning, Shanghai, China) (300 μl cell suspension, 1 × 10^5^ cells each chamber). The bottom compartment was filled with 600 μL of medium containing 10% FBS. The chambers were removed 48 h after incubation at 37°C. Then, the cells adhering to the membrane’s upper surface were removed, and the cells adhering to the membrane’s lower surface were fixed with anhydrous alcohol, and 0.1% crystal violet solution was utilized to stain them. Next, for each well, five randomly selected visual fields were photographed, and under the inverted microscope (Olympus, Tokyo, Japan), the cells were counted.

### Bioinformatics analysis and dual-luciferase reporter assay

The binding sites for miR-92a-3p in the BTG2 mRNA 3ʹUTR sequences were predicted with the StarBase database (http://starbase.sysu.edu.cn/). Mutant (MUT) 3ʹUTR of BTG2 sequence or wild type (WT) 3ʹUTR of BTG2 sequence was inserted into pGL3 promoter vectors (GenScript Co., Ltd., Nanjing, China). Subsequently, miR-92a-3p mimics and the vectors were co-transfected into MCF-7 and BT549 cells using Lipofectamine 2000 (Invitrogen, Carlsbad, CA, USA). The Dual-luciferase® Reporter Assay System (Promega Corp., Madison, WI, USA) was utilized to detect the relative luciferase activity of the cells 48 h after transfection.

### Western blot

RIPA lysis buffer (Beyotime, Shanghai, China) was applied for extracting the total protein from the cells. Subsequently, the total protein in each group was dissolved employing SDS-PAGE, and the protein was then transferred onto the PVDF membrane (Millipore, Billerica, MA, USA). Next, after being blocked with 5% bovine serum albumin at 37°C for 2 h, the membranes were incubated overnight at 4°C with the corresponding primary antibodies including anti-BTG2 (ab197362, 1:1000), anti-Bax (ab182733, 1:1000), anti-Bcl-2 (ab692, 1:1000) and anti-PPIA (ab126738, 1:1000). After washing the membranes three times with TBST, the membranes and horseradish peroxidase (HRP)-conjugated goat anti-rabbit (ab205718, 1:5000) or anti-mouse (ab6789, 1:5000) secondary antibodies were incubated at 37°C for 1 h. Ultimately, a Pierce ECL Western Blotting Substrate (Thermo Fisher Scientific, Inc., Waltham, MA, USA) was utilized for developing the bands. All of the antibodies were obtained from Abcam (Shanghai, China).

### Immunohistochemistry (IHC)

The paraffin sections were dewaxed and rehydrated. Then, the sections were heated (98°C) in 10 mM sodium citrate-hydrochloric acid buffer (pH 8.0) for 30 min for antigen retrieval, and incubated with 3% hydrogen peroxide for 30 min to inactivate endogenous peroxidase. Blocking solution was then added, and the unspecific antigens were blocked. Anti-BTG2 antibody (Abcam, ab197362, 1:100) was used to incubate the sections overnight at 4°C, and normal rabbit immunoglobulin G (IgG) functioned as the negative control. After the sections were washed, the goat anti-rabbit IgG (Abcam, ab205718, 1:100) secondary antibody was supplemented to incubate the sections at room temperature for 30 min. Then the slices were stained with DAB solution (Beyotime, Shanghai, China). Finally, the sections were observed and the staining was scored under a microscope. The scoring system was based on stained cells’ staining degree and percentage: 0, no coloring; 1, light brown; 2, dark brown; 0, stained cells < 5%; 1, stained cells between 5% and 25%; 2, stained cells between 26% and 50%; 3, stained cells > 50%. The final score was acquired via multiplying the scores of the staining degree and the percentage. The scores < 2: negative; scores ≥ 2 but < 4: weakly positive; scores ≥ 4: strongly positive. Normal IgG acted as the negative control in IHC.

### Statistical analysis

SPSS17.0 statistical software (SPSS Inc., Chicago, IL, USA) was the tool for statistical analysis, and mean ± standard deviation (x ± s) was adopted to represent the measurement data. The comparison between two groups was conducted through *t*-test, and one-way ANOVA was conducted for the comparison among multiple groups. χ^2^ was employed for analyzing the difference between enumeration data from two groups. The correlation between miR-92a-3p and BTG2 mRNA expressions were evaluated using Pearson’s correlation analysis. *P*< 0.05 implied that differences were of statistical significance.

## Results

The current research explored the expression and clinical significance of miR-92a-3p in BC, and further analyzed its effects on regulating BC cells’ multiplication, migration and invasion, and the underlying mechanism. This study showed that in BC tissues and cells, miR-92a-3p was highly expressed. MiR-92a-3p overexpression promoted the multiplication, migration and invasion of BC cells, while the opposite effect was observed in BC cells with down-regulated miR-92a-3p. Furthermore, BTG2 was a direct downstream miR-92a-3p target in BC cells. In addition, it was revealed that BTG2 counteracted the effects of miR-92a-3p on BC cell multiplication, migration and invasion.

### MiR-92a-3p expression and its clinical significance in BC

**Figure 1.**
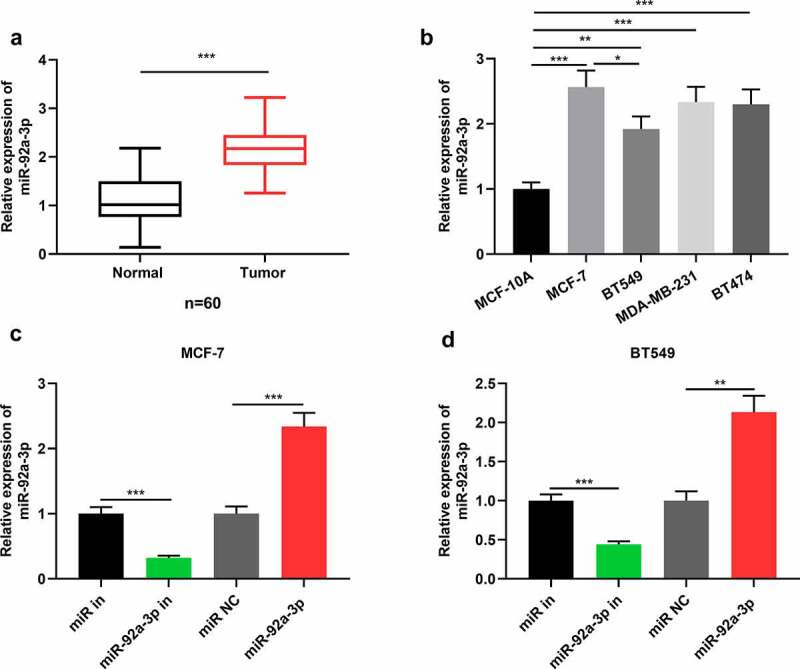
MiR-92a-3p is highly expressed in BC cells and tissues (a) Detection by qRT-PCR of relative miR-92a-3p expression in 60 adjacent normal tissues (normal) and 60 BC tissues (tumor). (b) Detection by qRT-PCR of relative miR-92a-3p expression in four kinds of BC cells (MCF-7, MDA-MB-231, BT549 and BT474) and normal breast epithelial cell line (MCF-10A). (c and d). MiR-92a-3p inhibitors (50 nM) or miR in (50 nM) and miR-92a-3p mimics (50 nM) or miR NC (50 nM) were transfected into MCF-7 or BT549 cells, respectively. And qRT-PCR was conducted to detect relative miR-92a-3p expression in MCF-7 and BT549 cells after the transfection

First of all, miR-92a-3p expression in BC tissues and cell lines was detected via qRT-PCR, and it was demonstrated that in comparison to normal tissues, miR-92a-3p in BC tissues was markedly up-regulated (Figure 1(a)). Meanwhile, miR-92a-3p expression was enhanced in four kinds of BC cell lines in comparison to the cell line MCF-10A (Figure 1(b)). Since miR-92a-3p expression is the highest in MCF-7 cells and the lowest in BT549 cells, and the difference of miR-92a-3p expression in BT549 and MCF-7 cells is statistically significant (Figure 1(b)). Therefore, we used BT549 and MCF-7 cells for further research. Subsequently, miR-92a-3p mimics and inhibitors were transfected into BT549 and MCF-7 cells, respectively, and qRT-PCR showed that miR-92a-3p inhibitors reduced miR-92a-3p expression in BT549 and MCF-7 cells, while miR-92a-3p mimics could increase it (Figure 1(c,d)). Additionally, to clarify the clinical significance of highly expressed miR-92a-3p in BC progression, chi-square test was utilized for analyzing the correlation between miR-92a-3p expression and cliniopathological parameters of BC, and it suggested that high miR-92a-3p expression was associated with larger tumor size and increased TNM stage of the patients (Table 1).

The experiments were performed in triplicate. **P< 0.01, ***P< 0.001 vs control/normal group, MCF-10A cells, miR in or miR NC.

### MiR-92a-3p facilitates BC cell multiplication and metastasis

**Figure 2.**
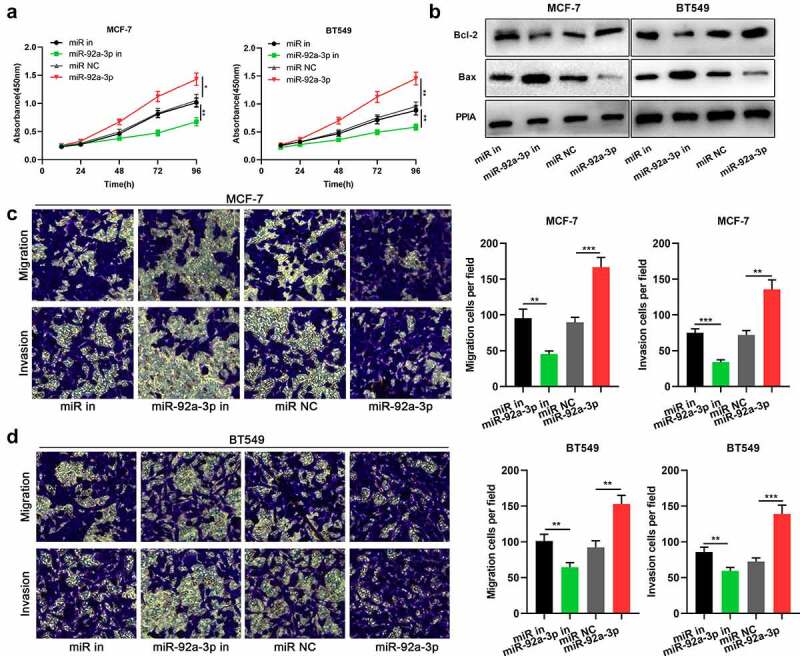
MiR-92a-3p promotes BC cell proliferation and metastasis (a) The proliferation of BT549 and MCF-7 cells transfected with 50 nM miR-92a-3p mimics (or 50 nM miR NC) and 50 nM miR-92a-3p inhibitors (or 50 nM miR in) was detected by CCK-8 assay. (b) Western blot was utilized for examining the expression levels of apoptosis-related proteins (Bcl-2/Bax) in BT549 and MCF-7 cells transfected with 50 nM miR-92a-3p mimics (or 50 nM miR NC) and 50 nM miR-92a-3p inhibitors (or 50 nM miR in). (c and d). Transwell assays were employed for detecting the invasion and migration of BT549 and MCF-7 cells transfected with 50 nM miR-92a-3p mimics (or 50 nM miR NC) and 50 nM miR-92a-3p inhibitors (or 50 nM miR in)

After confirming that miR-92a-3p was highly expressed in BC, we then explored its functions in BC cells. CCK-8 assay manifested that miR-92a-3p inhibition repressed MCF-7 and BT549 cells’ proliferation, whereas the transfection of miR-92a-3p mimics facilitated MCF-7 and BT549 cells’ proliferation (Figure 2(a)). Western blot showed that miR-92a-3p inhibition facilitated Bax expression and suppressed Bcl-2 expression, while miR-92a-3p overexpression suppressed Bax expression and facilitated Bcl-2 expression (Figure 2(b)). Moreover, through Transwell assay, it was discovered that miR-92a-3p inhibition restrained MCF-7 and BT549 cells’ migration and invasion, while the transfection of miR-92a-3p mimics had opposite effects (Figure 2(c,d)).

The experiments were performed in triplicate. *P< 0.05, **P< 0.01 and ***P< 0.001 vs. miR in or miR NC group.

### BTG2 is the target of miR-92a-3p

**Figure 3.**
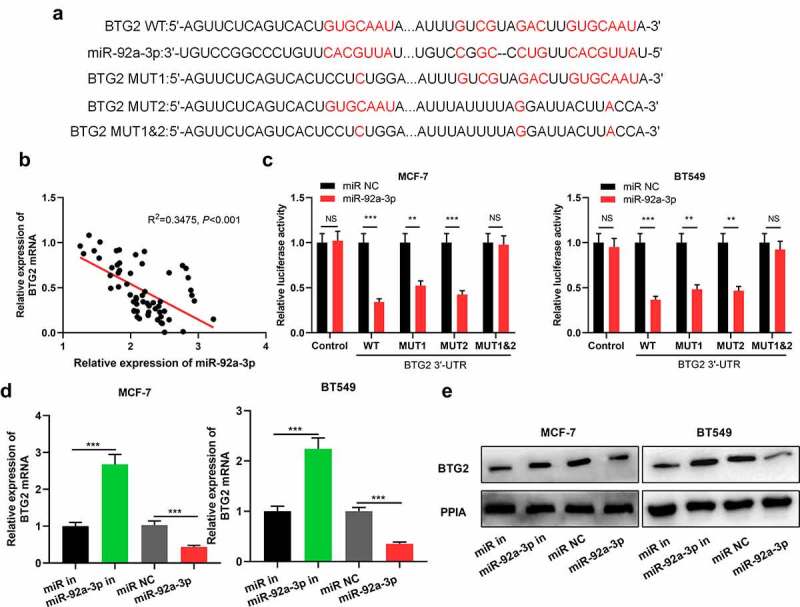
BTG2 is a miR-92a-3p target (a) The binding sequence of miR-92a-3p with BTG2 was predicted through Bioinformatics analysis. (b) Pearson correlation analysis was conducted to analyze the correlation between miR-92a-3p expression and BTG2 mRNA expression in 60 BC tissues. (c) Dual-luciferase reporter gene assay was utilized for detecting the luciferase activity of BTG2 3ʹUTR WT or MUT after miR-92a-3p was overexpressed. D and E. Western blot and qRT-PCR were carried out to examine BTG2 mRNA and protein expressions in BT549 and MCF-7 cells with transfection of 50 nM miR-92a-3p mimics (or 50 nM miR NC) and 50 nM miR-92a-3p inhibitors (or 50 nM miR in)

To decipher the mechanism by which miR-92a-3p exerted its biological function, we searched StarBase database for miR-92a-3p targets, and it showed that there were potential binding sites between BTG2 3ʹ-UTR and miR-92a-3p (Figure 3(a)). Besides, qRT-PCR displayed that in BC tissues, miR-92a-3p and BTG2 mRNA expression levels were negatively correlated (Figure 3(b)). Then, dual-luciferase reporter gene assay was conducted for verifying the predicted binding sites between miR-92a-3p and BTG2. It was suggested that as opposed to the miR NC group, up-regulation of miR-92a-3p markedly suppressed BTG2 3ʹUTR WT’s luciferase activity, but could not affect BTG2 3ʹUTR MUT’s luciferase activity (Figure 3(c)). Furthermore, it was discovered that miR-92a-3p inhibitors facilitated BTG2 mRNA and protein expression in BT549 and MCF-7 cells, while its mimics produced the opposite effect in MCF-7 and BT549 cells (Figure 3(d,e)). The aforementioned findings reveal that miR-92a-3p directly targets BTG2 to reduce its expression in BC.

The experiments were performed in triplicate. **P< 0.01 and ***P< 0.001, NS, *P*> 0.05 vs miR NC or miR in group.

### BTG2 is lowly expressed in BC cells and tissues

**Figure 4.**
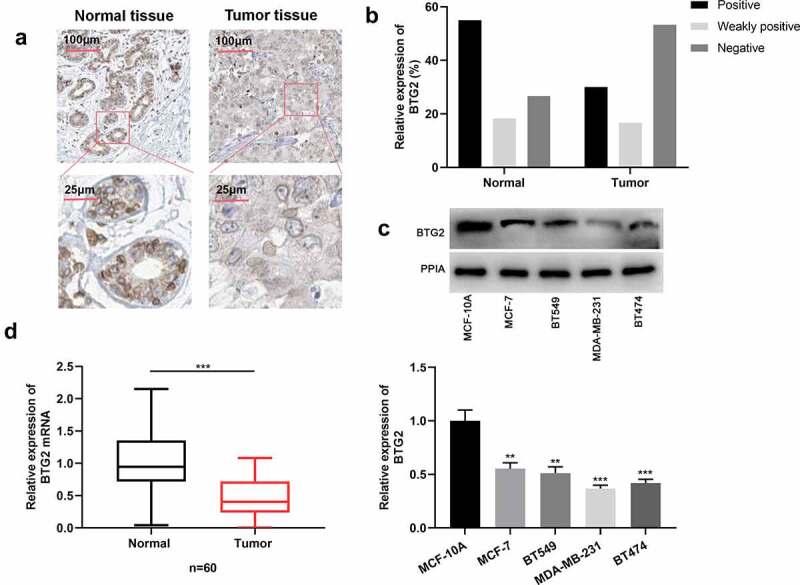
BTG2 is lowly expressed in BC (a) IHC was adopted to examine BTG2 expression in para-cancerous tissues and cancerous tissues. (b) The strongly positive, weakly positive and negative expression rates of BTG2 in adjacent tissues and BC tissues were obtained through statistics. (c) Western blot was performed to detect BTG2 expression in BC cells (MDA-MB-231, BT549, MCF-7 and BT474) and normal breast epithelial cell line MCF-10A. (d). BTG2 mRNA expression in adjacent normal tissues (normal) and BC tissues (tumor) was examined by qRT-PCR

Next, BTG2 expression characteristics in BC was investigated. IHC was employed to determine BTG2 expression level in cancerous tissues and normal adjacent tissues of 60 BC patients. The statistical results exhibited that in para-cancerous tissues, the strongly positive expression rate of BTG2 was 55%, the weakly positive expression rate 18.33%, and the negative expression rate 26.67%, while in BC tissue samples, its strongly positive expression rate was 30%, the weakly positive expression rate 16.67%, and the negative expression rate 53.33%. This suggested that in comparison to adjacent normal tissues, BTG2 is down-regulated in BC tissues (Figure 4(a,b)). Subsequently, BTG2 expression in BC cells was detected by Western blot, and it indicated that BTG2 expression in BC cells was remarkably down-regulated (Figure 4(c)). In addition, qRT-PCR showed that compared to normal tissues, BTG2 mRNA expression was reduced in BC tissues (Figure 4(d)).

The experiments were performed in triplicate. **P< 0.01 and ***P< 0.001 vs control/normal group or MCF-10A cells.

### MiR-92a-3p regulated BC cell metastasis and proliferation by targeting BTG2

**Figure 5.**
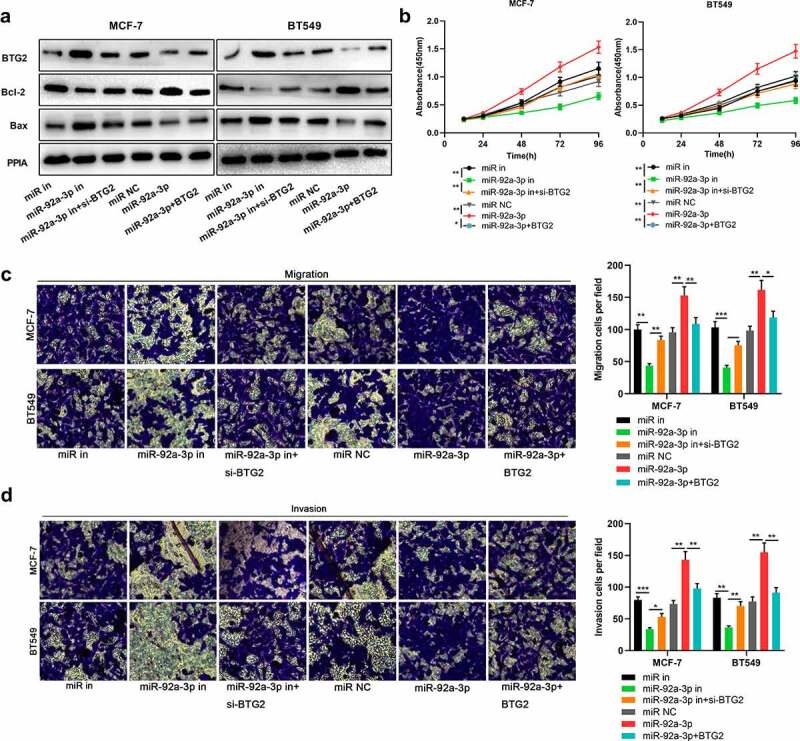
MiR-92a-3p/BTG2 axis participates in regulating BC cell metastasis and proliferation. 50 nM BTG2 siRNA (si-BTG2) and miR-92a-3p inhibitors were co-transfected into MCF-7 and BT549 cells, and BT549 and MCF-7 cells were also co-transfected with BTG2 overexpression plasmids and miR-92a-3p mimics.(a). BTG2, Bcl-2 and Bax protein expression in BT549 and MCF-7 cells was detected by Western blot, and PPIA protein served as the endogenous control. (b). CCK-8 assay was utilized for detecting MCF-7 and BT549 cells’ proliferation after co-transfection. (c and d). Transwell assays were employed for detecting BT549 and MCF-7 cells’ invasion and migration after co-transfection

To delve into miR-92a-3p/BTG2 axis’s role in BC, we transfected BTG2 siRNA into BT549 and MCF-7 cells with miR-92a-3p inhibition, and transfected BTG2 overexpression plasmids into BT549 and MCF-7 cells with miR-92a-3p overexpression, and then BTG2, Bcl-2 and Bax protein expression levels were detected through Western blotting. It was unveiled that knocking down BTG2 reversed the promoting effects of transfection of miR-92a-3p inhibitors on BTG2 and Bax protein levels in BT549 and MCF-7 cells, as well as the down-regulation of Bcl-2 protein level (Figure 5(a)); BTG2 overexpression reversed the inhibiting effect that up-regulation of miR-92a-3p had on BTG2 and Bax protein levels in BT549 and MCF-7 cells, and the promotion of Bcl-2 protein level (Figure 5(a)). It was also found that BTG2 interference partially counteracted the inhibiting impact of transfection of miR-92a-3p inhibitors on MCF-7 and BT549 cells’ proliferation, migration and invasion (Figure 5(b-d)). Meanwhile, overexpression of BTG2 weakened the promoting effect of the miR-92a-3p mimics transfection on MCF-7 and BT549 cells’ proliferation, migration and invasion (Figure 5(b-d)). The aforementioned findings show that the miR-92a-3p/BTG2 axis takes part in modulating BC cell proliferation and metastasis.

The experiments were performed in triplicate. *P< 0.05, **P< 0.01 and ***P< 0.001 vs miR in, miR-92a-3p in+si-BTG2, miR NC or miR-92a-3p+BTG2 group.

## Discussion

The dysregulated miRNAs functions as tumor suppressors or oncogenic factors in BC progression. For instance, miR-185-5p reportedly regulates RAGE to inhibit actin polymerization and reverse the epithelial-mesenchymal transition of BC cells, and miR-185-5p down-regulation is associated with unfavorable clinicopathological features of BC patients, which implies a poor prognosis [17]. What’s more, miR-21 is markedly highly expressed in BC, and can function as a biomarker for BC diagnosis, and it promotes BC cell proliferation and metastasis via targeting LZTFL1 [18]. MiR-92a-3p plays a cancer-promoting part in a series of human tumors [9,10,19–22]. In colorectal cancer cells, miR-92a-3p served as an oncomiR by modulating PTEN/PI3K/AKT pathway [20]. MiR-92a also activates the JNK signaling via targeting DUSP10 and is involved in regulating pancreatic cancer cell proliferation [21]. Besides, miR-92a-3p has been reported to be increased in BC [22], and this is in line with our research results. In this work, the cancer-promoting effect of miR-92a-3p in BC was further confirmed. It was revealed that high miR-92a-3p expression was linked to increased TNM stage and large tumor size. In terms of function, it was validated that miR-92a-3p inhibitors enhanced the pro-apoptotic protein Bax expression, and suppressed cell multiplication, metastasis and the expression of apoptosis-inhibitory protein Bcl-2, while transfection of miR-92a-3p mimics inhibited Bax, and facilitated cell proliferation, metastasis and Bcl-2 expression. The above-mentioned evidence suggests that miR-92a-3p can probably function as a predictor for the BC patient’s poor prognosis and a potential treatment target.

BTG2 is a member of the BTG/Tob anti-proliferative protein family. There is a conserved domain at the N terminus, the BTG domain, which characterizes BTG/Tob factors [23,24]. BTG2 is expressed in diverse tissues and organs, e.g., lung tissue, thymus, spleen and gastrointestinal tissue, and participates in multiple biological processes, e.g., cell proliferation, differentiation, DNA damage repair and apoptosis [23,25]. Previous studies have reported that BTG2 is lowly expressed in many malignancies, e.g., lung cancer, hepatocellular carcinoma and gastric cancer [12–14], and functions as a possible target for malignancy prevention and treatment. BTG2 is down-regulated in hepatocellular carcinoma and its low expression is related to poor clinicopathological features, and participates in the inhibition of cancer stem cell-like features of side population cells [13]. BTG2 can also inhibit osteosarcoma cell multiplication and metastasis via restraining PI3K/AKT pathway [26]. Furthermore, BTG2 is demonstrated to be significantly lowly expressed in BC, and its overexpression suppresses MDA-MB-231 cell line multiplication and invasion, and facilitates apoptosis [15,16]. Another study demonstrates that BTG2 suppresses BC progression by differentially regulating mTORc2-AKT1-NFAT1-PHLPP2 and mTORc1 signal axes [27]. These studies indicates that BTG2 can suppress BC via multiple signal pathways. In this work, we validated that BTG2 expression level was reduced in BC tissues, which is in line with its tumor-suppressive function reported in previous reports [15,16,27].

It is well known that miRNAs play their roles mainly based on targeting downstream genes [17,18,21]. Previous reports have shown that miR-92a-3p has multiple target genes, most of which are tumor suppressors, including PTEN, MYCBP2, FBXW7 and so on [9,19,28]. To further decipher the downstream mechanism of miR-92a-3p in BC progression, in this work, bioinformatics was utilized for predicting the potential miR-92a-3p target genes (Supplementary Materials), and BTG2 was obtained. Interestingly, BTG2 and miR-92a-3p expressions were inversely correlated in BC tissues, which also hinted a regulatory relationship between them. Next, dual-luciferase reporter gene analysis proved that there existed a targeted relationship between miR-92a-3p and BTG2. In addition, miR-92a-3p inhibition could greatly increase BTG2 expression at the levels of both protein and mRNA, whereas the miR-92a-3p mimics transfection suppressed BTG2 expression. More importantly, knockdown of BTG2 counteracted the inhibiting impact that miR-92a-3p inhibitors had on the malignant biological behaviors of BC cells, while overexpression of BTG2 partially attenuated the cancer-promoting effect of miR-92a-3p in BC. These data not only clarify the downstream mechanism of miR-92a-3p, but also partly explained the mechanism of BTG2 dysregulation in BC. Taken together, our research shows that miR-92a-3p can facilitate BC cell multiplication, migration and invasion via down-regulating BTG2 (Graphical abstract).

## Conclusion

In conclusion, miR-92a-3p expression is elevated in BC tissues and cells. It facilitates BC development through regulating BTG2. The finding of the miR-92a-3p/BTG2 axis may present an effective clinical therapeutic strategy for BC patients. Nevertheless, in the future, it is still necessary to perform *in vivo* studies to further verify the demonstrations in this research, and other miR-92a-3p target genes in BC remains to be screened and validated.

## Supplementary Material

Supplemental Material

## Article highlights

1. The expression of miR-92a-3p is up-regulated in BC tissues and cell lines.
2. The high expression of miR-92a-3p is associated with larger tumor size of BC patients and the increase of clinical stage.
3. miR-92a-3p promotes the proliferation, migration and invasion of BC cells.

miR-92a-3p can directly target BTG2 and suppress its expression.
