# Supplementary material for: MicroRNA miR-92a-3p regulates breast cancer cell proliferation and metastasis via regulating B-cell translocation gene 2 (BTG2)
Source: Bioengineered. 2021 Jun 3;12(1):2033–44. doi: 10.1080/21655979.2021.1924543 (PMC8806219; doi:10.1080/21655979.2021.1924543)

**The original image of Western blot experiment**

Figure 2 B MCF-7

Maker negative/positive control Bcl-2


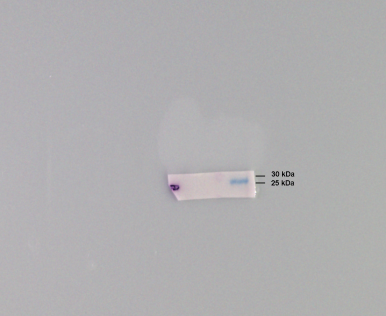

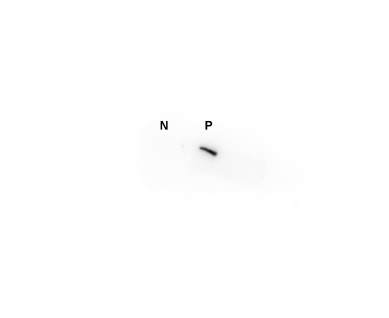

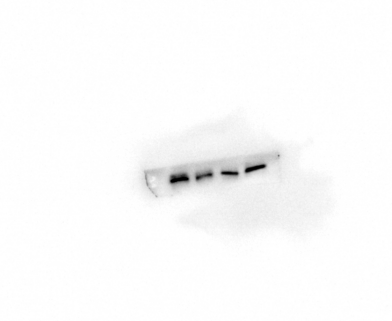


Maker negative/positive control Bax


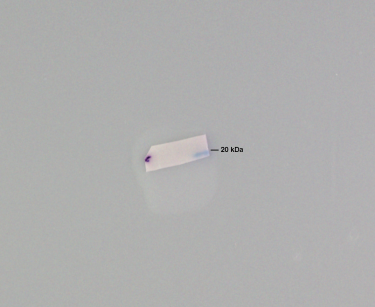

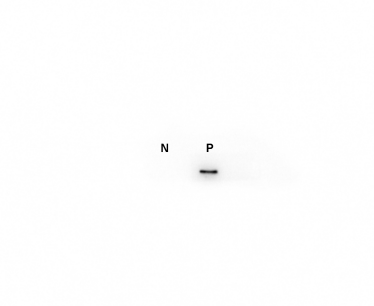

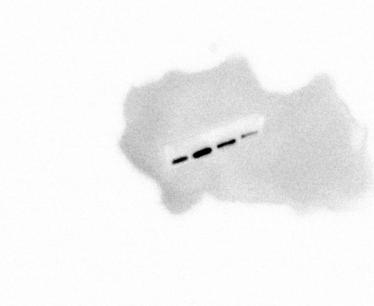


Maker negative/positive control PPIA


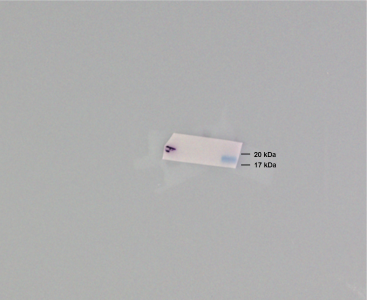

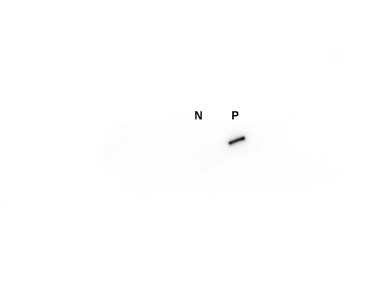

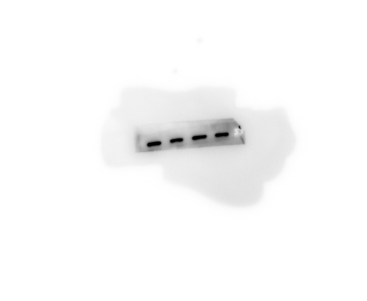


Figure 2 B BT549

Maker negative/positive control Bcl-2


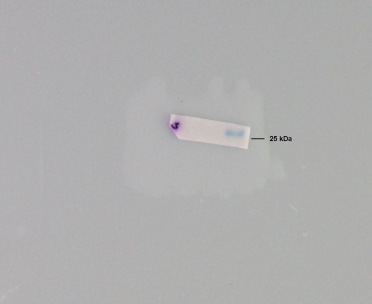

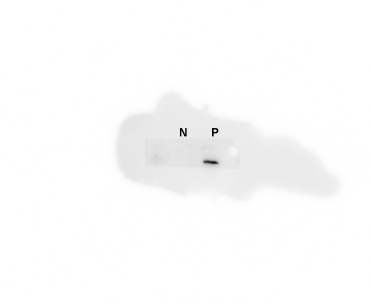

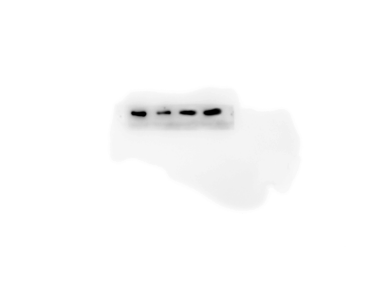


Maker negative/positive control Bax


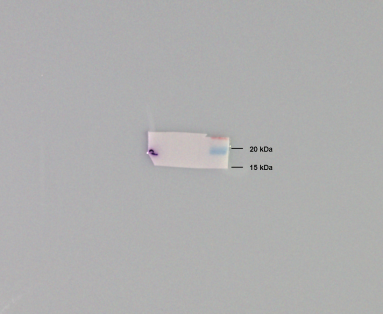

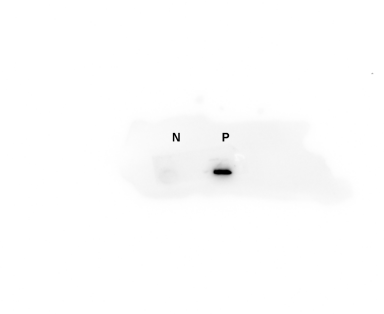

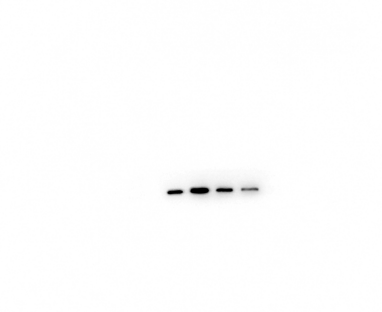


Maker negative/positive control PPIA


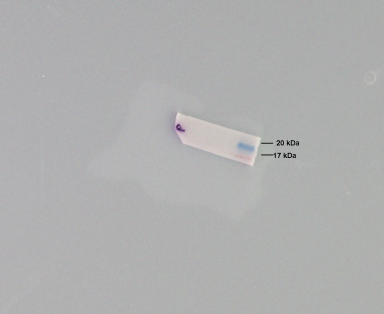

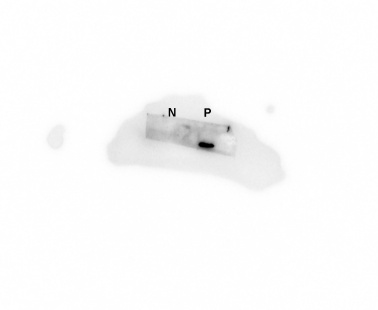

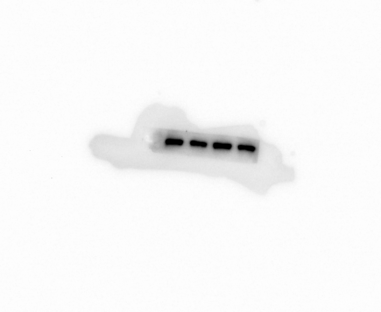


Figure 3 E MCF-7

Maker negative/positive control BTG2


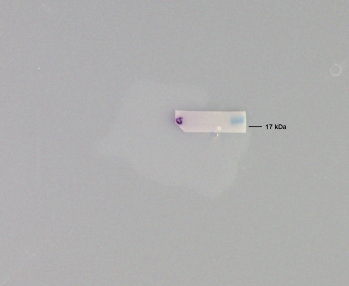

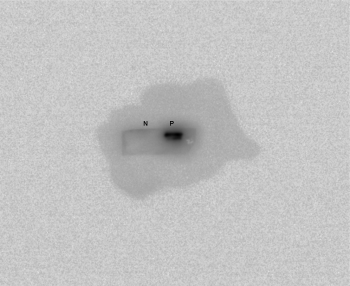

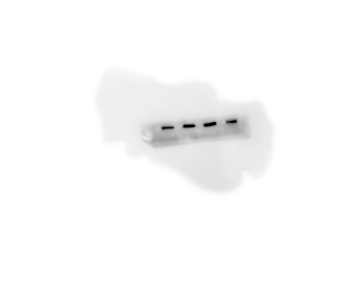


Maker negative/positive control PPIA


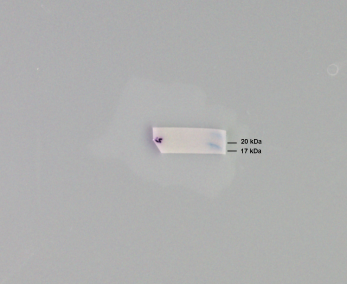

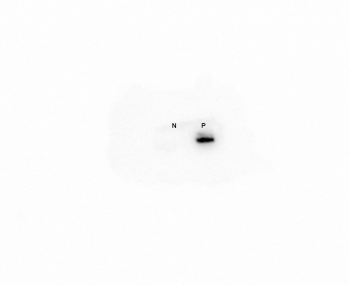

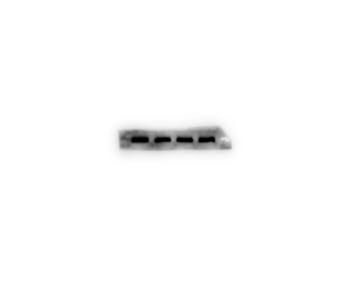


Figure 3 E BT549

Maker negative/positive control BTG2


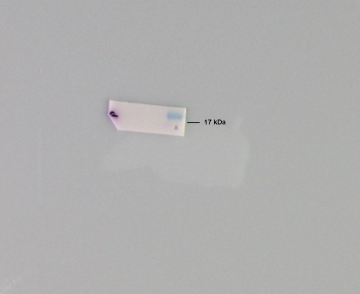

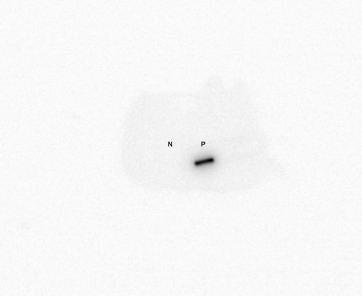

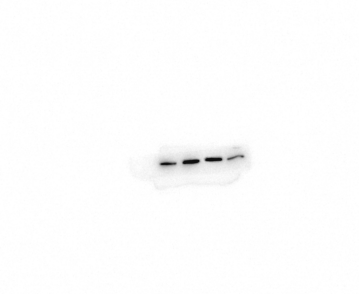


Maker negative/positive control PPIA


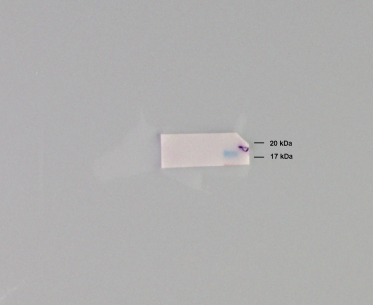

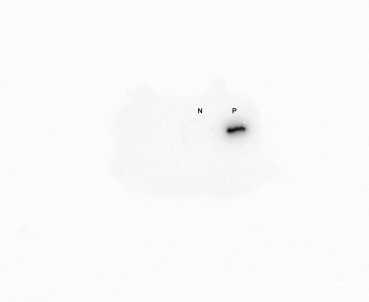

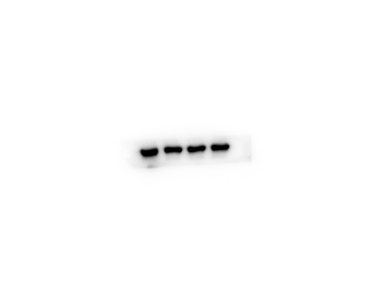


Figure 4 C

Maker negative/positive control BTG2


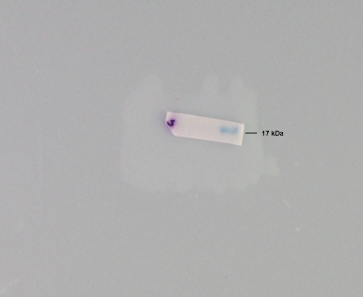

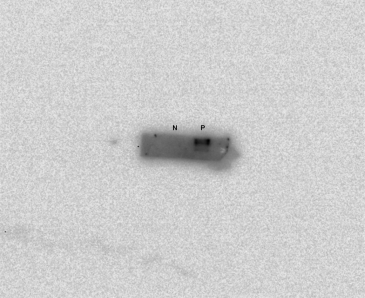

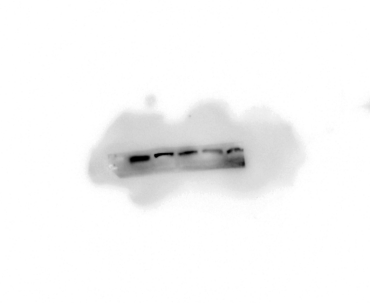


Maker negative/positive control PPIA


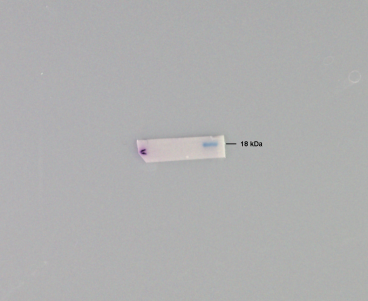

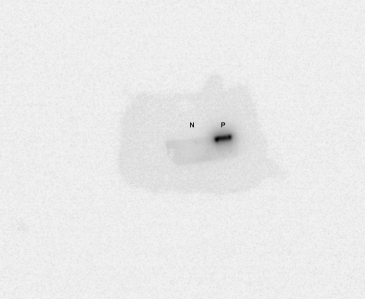

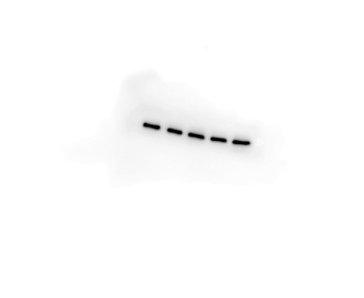


Figure 5 A MCF-7

Maker negative/positive control BTG2


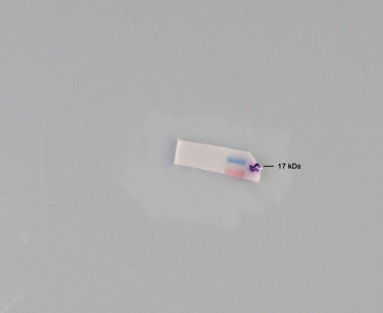

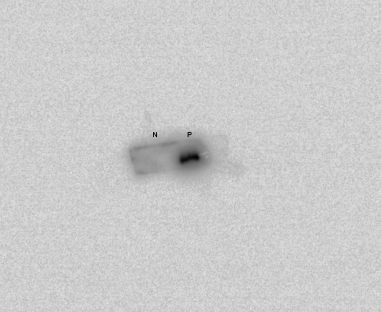

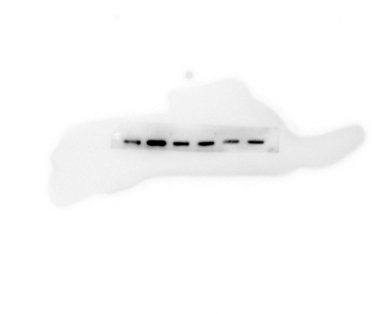


Maker negative/positive control Bcl-2


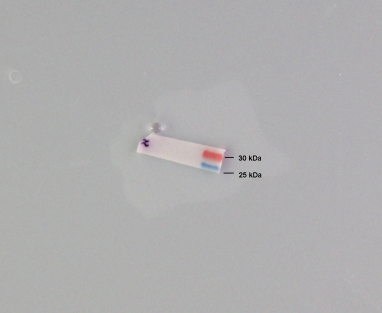

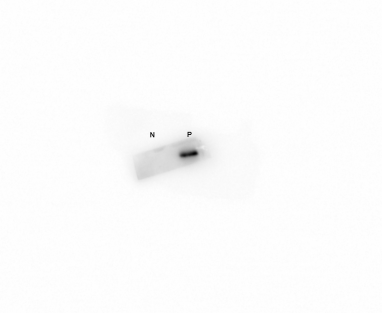

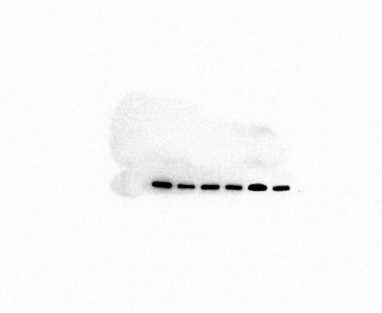


Maker negative/positive control Bax


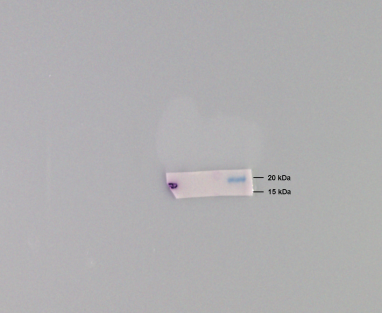

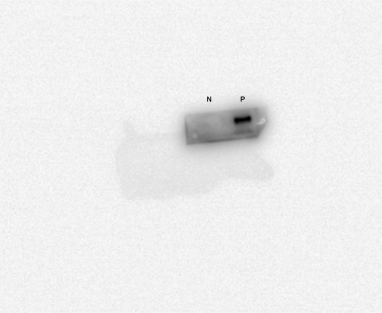

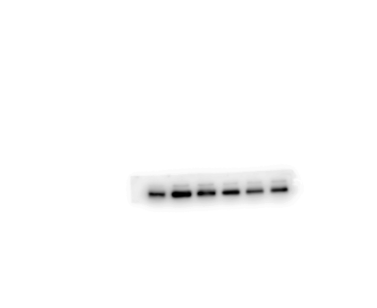


Maker negative/positive control PPIA


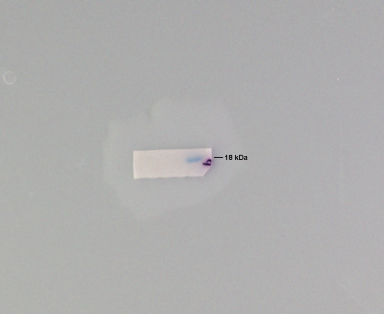

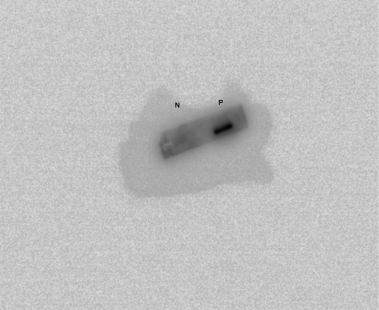

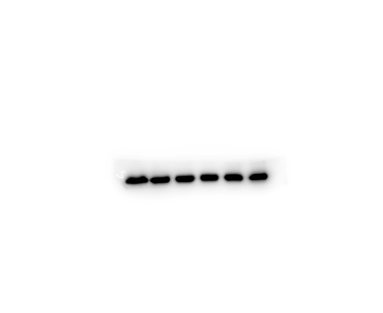


Figure 5 A BT549

Maker negative/positive control BTG2


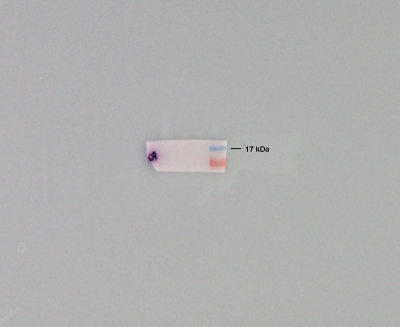

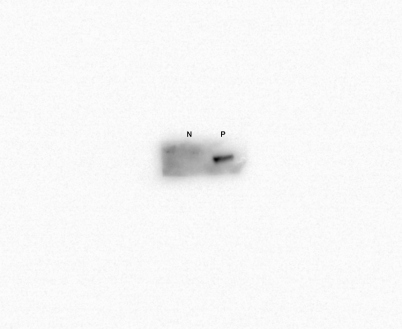

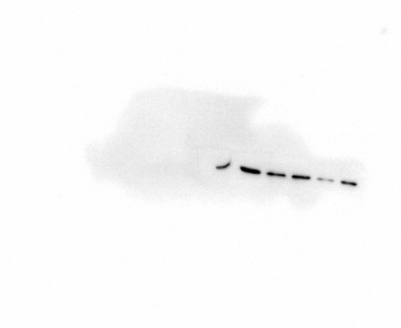


Maker negative/positive control Bcl-2


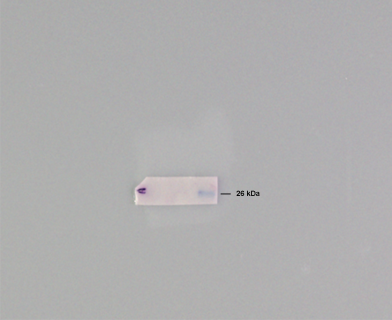

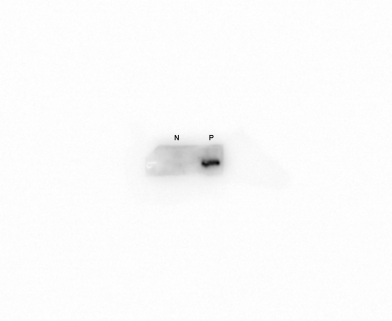

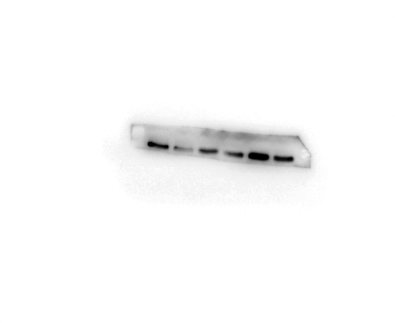


Maker negative/positive control Bax


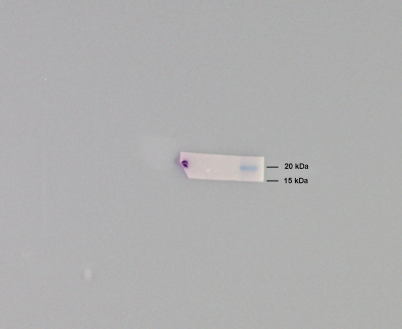

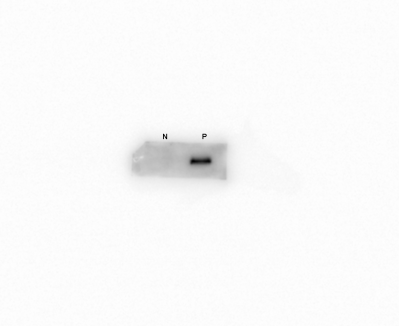

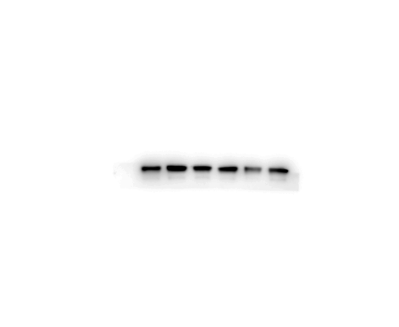


Maker negative/positive control PPIA


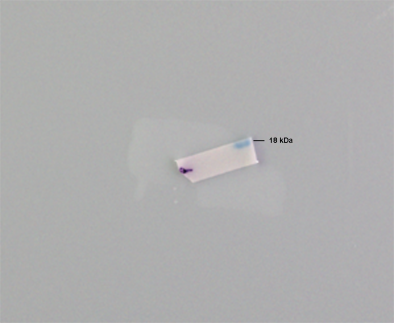

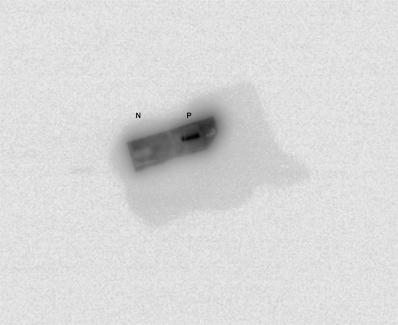

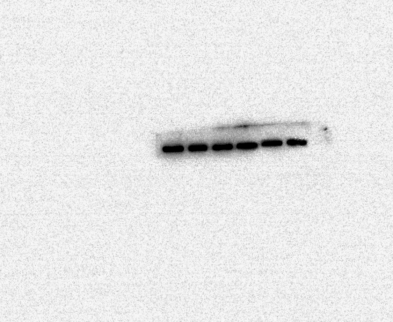

Supplement: Supplemental Material [file KBIE_A_1924543_SM9236.zip › Supplementary data_orignal Western blot data_only for reviewing.docx]
